# Ogilvie syndrome in a 8 year old girl after laparoscopic appendectomy

**DOI:** 10.1186/s12887-019-1457-z

**Published:** 2019-03-20

**Authors:** Giulia Gortani, Federica Pederiva, Lydie Ammar, Elisabetta Miorin, Giovanna Tonin, Giulia Dobbiani, Elena Marcuzzi, Egidio Barbi

**Affiliations:** 1 Pediatrics, Institute for Maternal and Child Health - IRCCS “Burlo Garofolo”, Trieste, Italy; 2 Pediatric Surgery, Institute for Maternal and Child Health, IRCCS “Burlo Garofolo”, Via dell’Istria 65/1, 34137 Trieste, Italy; 3 Radiology, Institute for Maternal and Child Health, IRCCS “Burlo Garofolo”, Trieste, Italy; 4 Pediatric Care Unit, Hospital of Palmanova, Udine, Italy; 5 University of Trieste, Trieste, Italy

**Keywords:** Ogilvie’s syndrome, Acute colonic pseudo-obstruction, Case report

## Abstract

**Background:**

Ogilvie’s syndrome is described in the adult population, but rarely seen in children.

**Case presentation:**

We present a case of a girl who suffered acute colonic pseudo-obstruction after laparoscopic appendectomy.

**Conclusions:**

Ogilvie’s syndrome, although rare in the pediatric population, should be considered as possible diagnosis after a surgical procedure in presence of persisting subocclusive symptoms and radiological signs of massive colonic dilatation without mechanical obstruction.

## Background

Ogilvie’s syndrome is a rare complication mostly described in adult patients with severe underlying medical or surgical conditions [1]. However, an increased number of cases of Ogilvie’s syndrome has been reported in children with oncological diseases [2–5], after spinal surgery [6], in sickle cell and Kawasaky disease [7, 8], and after renal transplant [9].

We present a case of a girl who suffered acute colonic pseudo-obstruction after laparoscopic appendectomy.

## Case presentation

An 8-year-old girl, who had a laparoscopic appendectomy for gangrenous appendicitis at another hospital, presented with postoperative persisting non-bilious vomiting and progressive abdominal distension initially diagnosed as paralytic ileus and treated with conservative management. Despite this, her clinical condition did not improve; she passed spontaneously flatus, but she couldn’t have bowel movements without administration of enemas. Laboratory findings were within normal range. Ten days later, as the clinical conditions failed to improve, she was transferred to our hospital.

On physical examination at admission, the abdomen was distended and tympanitic to percussion, but soft with no tenderness, rebound or guarding. Bowel sounds were present. Laboratory findings were within normal range and no free fluid or collections were found at the US scan.

The abdominal x-ray showed marked colonic gaseous dilatation, without evidence of mechanical obstruction. Gut decontamination with oral ciprofloxacin and metronidazole was started and oral intake was progressively resumed.

**Fig. 1:**
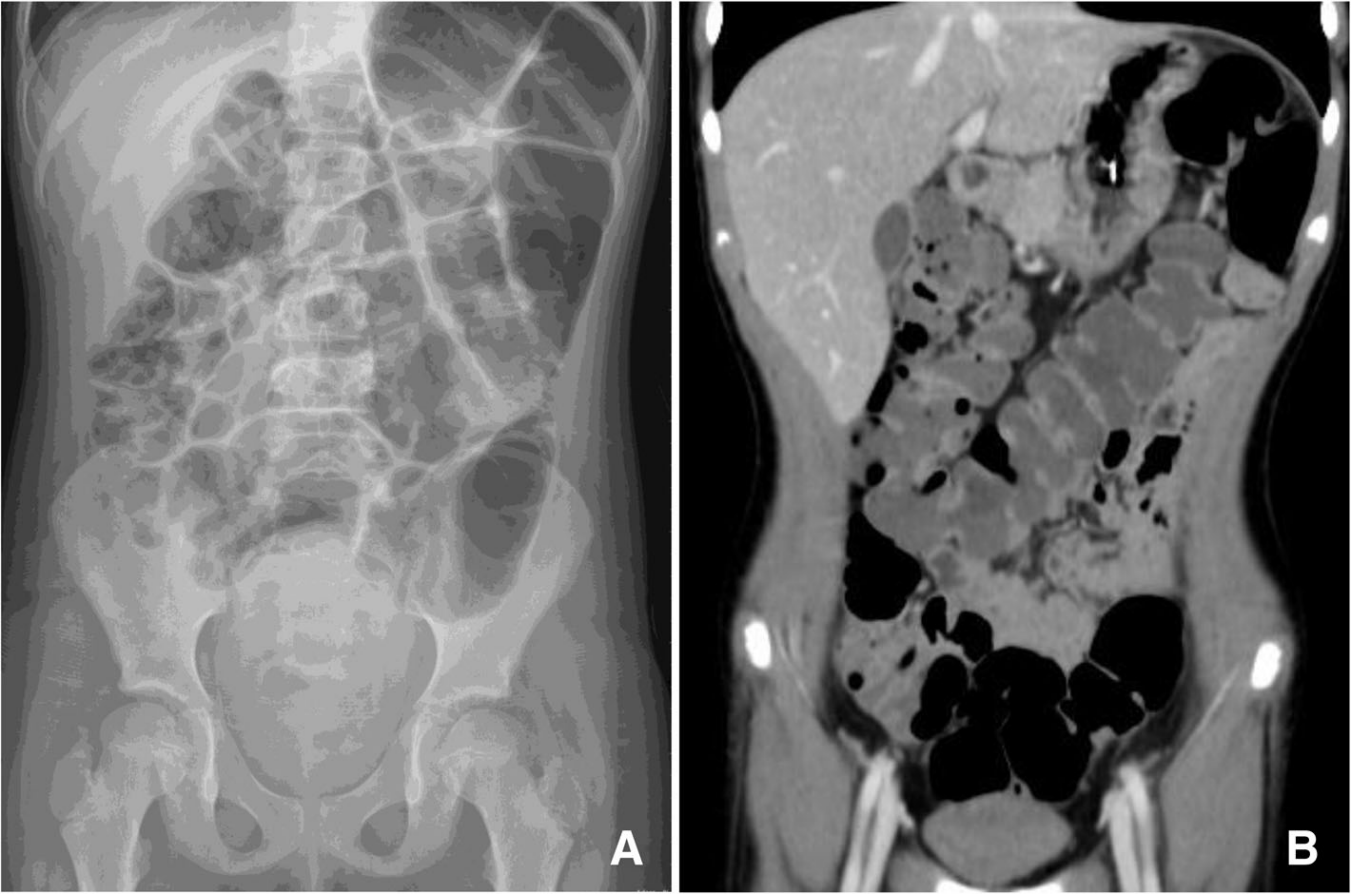
**a** Anterioposterior abdominal x-ray showing marked dilatation of the colon, without air/fluid levels and free air. **b** Coronal computed tomography revealed massive dilatation of the large bowel with minimal involvement of the small bowel

The symptoms improved temporarily, but, unfortunately, they recurred 4 days after admission with greater severity in presence of bilious vomiting. The abdominal x-ray showed worsening colonic dilation (Fig. 1a) and rectal stool impaction. Because of the diminished bowel sounds, an abdominal computed tomography scan was performed and confirmed the severe colonic dilatation form the cecum to the splenic flexure in absence of colonic mechanical obstruction (Fig. 1b). A nasogastric tube and a rectal tube were inserted to put bowel at rest, parenteral nutrition was started, the patient was kept on nil by mouth and a combination of stool softeners and enemas were administered. The clinical and radiological findings suggested the diagnosis of Ogilvie’s syndrome. Erythromicyn (250 mg two times a day) through the nasogastric tube was then started followed by an improvement in clinical symptoms. The medication was stopped after seven days. The patient made a full recovery, resuming her normal diet and spontaneous bowel movements, and was discharged in good conditions 25 days after the surgery. At the follow up appointment one week after discharge she was well and completely recovered.

## Discussion

Ogilvie’s syndrome, or acute colonic pseudo-obstruction, is characterized by decreased gastrointestinal motility, massive dilatation of the colon without signs of mechanical obstruction, and limited small bowel involvement, otherwise affected in postoperative adynamic ileum. It occurs primarily in adult patients, whereas it happens rarely in children. It has been suggested that it could be the result of suppression of sacral parasympathetic nerves’ activity together with an increase in sympathetic impulses, resulting in inhibition of colonic motility and hence in colonic dilatation [5, 6, 10]. Other pathogenetic theories include prostaglandin E abnormalities, decreased splanchnic perfusion, side effects of neurotropic medications, metabolic disorders affecting neuromuscular conduction (hypokalaemia, uremia) and herpes zoster virus reactivation [10]. Patients present with recurrent vomiting, nausea, abdominal pain and prominent abdominal distension [6].

The syndrome tends to occur in adult patients with serious medical and surgical conditions, as myocardial infarction, neoplasia, metabolic disorders, spinal injury, peritonitis, sepsis and shock. In pediatric population it has been described in oncology patients, especially when neutropenic and treated with high-dose vincristine [2–5] in patients who underwent spinal surgery [6], after administration of anticholinergic medication [11], in sickle cell disease [7], in Kawasaky disease [8], and after renal transplant [9].

There are no pathognomonic laboratory tests, but the abdominal x-ray reveals large bowel dilatation, which especially involves cecum and ascending colon, with preservation of haustral markings and with little fluid in the bowel lumen, in contrast to mechanical obstruction, which contains multiple air/fluid levels. Computed tomography imaging is more specific than plain radiography to exclude any cause of mechanical obstruction and often reveals proximal colonic dilatation with a transitional zone adjacent to the splenic flexure.

Prompt diagnosis is essential to prevent the risk of ischemia, necrosis and, ultimately, perforation. Early management of Ogilvie’s Syndrome is conservative and includes observation of the patient on bowel rest and nil by mouth, parenteral nutrition, correction of fluid and electrolyte imbalances, bowel decompression via nasogastric suction and rectal tube, administration of enemas and promotility agents, limitation of narcotic medication. Close monitoring with frequent physical examination and eventually repeating of abdominal x-ray are recommended in order to highlight changes in clinical condition. If supportive therapy fails, once bowel perforation or ischemia have been ruled out, medical therapy might be implemented. The motilin agonist erythromycin stimulates gastric and small bowel motor activity and induces smooth muscle contraction through a nifedipine-sensitive mechanism. Given its use in children with gastrointestinal dysmotility, it may be a safe choice for treatment of Ogilvie’s syndrome. However, the short half-life of erythromycin and the rapid onset of tachyphylaxis might preclude its broad administration in this syndrome [7, 12], with some evidence suggesting a limited efficacy on colonic motility [13].

The parasympathomimetic agent neostigmine has been proved successful in treatment of Ogilvie’s syndrome in adults and had been used in few cases also in children. Through stimulation of the parasympathetic nervous system, it increases colonic contractility. The side effects of cholinesterase inhibitors include hypersalivation, nausea, vomiting, abdominal pain, bradycardia, hypotension and bronchospasm. During the infusion of neostigmine, patients should be monitored for possible development of bradycardia or bronchospasm [4, 5, 7, 14].

In this case we empirically preferred to use Erythromicin rather than neostigmine due to the better safety record and ease of use, even in front of a controversial literature on its efficacy. Limited evidence in the literature suggests that amoxicillin-clavulanate could be considered as an alternative [15].

If the conservative treatment failed to succeed, more aggressive intervention, such as colonoscopic decompression or surgery, are required to prevent perforation [4, 6, 10].

In our patient the diagnosis had not been immediately suspected as paralytic ileum was the more obvious diagnosis after a surgical procedure. However, the scarce involvement of the ileus and the massive colonic dilatation, not typical of adynamic ileum, had suggested the correct diagnosis.

## Conclusions

Ogilvie’s syndrome is a rare condition in the pediatric population. It mainly occurs in complex medical and surgical patients, but it should be considered as possible diagnosis after any surgery in case of persisting subocclusive symptoms with radiological signs of massive colonic dilatation in absence of mechanical obstruction.

## Abbreviations

mg: milligrams
US: Ultrasound
